# Surface displaced alfa-enolase of *Lactobacillus plantarum *is a fibronectin binding protein

**DOI:** 10.1186/1475-2859-8-14

**Published:** 2009-02-16

**Authors:** Cristiana Castaldo, Valeria Vastano, Rosa Anna Siciliano, Marco Candela, Manuela Vici, Lidia Muscariello, Rosangela Marasco, Margherita Sacco

**Affiliations:** 1 Dipartimento di Scienze Ambientali, Seconda Università di Napoli, via Vivaldi 43, 81100 Caserta, Italy; 2 Centro di Spettrometria di Massa Proteomica e Biomolecolare, Istituto di Scienze dell'Alimentazione, CNR, Avellino, Italy; 3 Dipartimento di Scienze Farmaceutiche, Università di Bologna, via Belmeloro 6, 40126 Bologna, Italy; 4 Dipartimento di Patologia Sperimentale, Università di Bologna, via S. Giacomo 14, 40126 Bologna, Italy; 5 Dipartimento di Scienze della Vita, Seconda Università di Napoli, via Vivaldi 43, 81100 Caserta, Italy

## Abstract

**Background:**

Lactic acid bacteria of the genus *Lactobacillus *and *Bifidobacterium *are one of the most important health promoting groups of the human intestinal microbiota. Their protective role within the gut consists in out competing invading pathogens for ecological niches and metabolic substrates. Among the features necessary to provide health benefits, commensal microorganisms must have the ability to adhere to human intestinal cells and consequently to colonize the gut. Studies on mechanisms mediating adhesion of lactobacilli to human intestinal cells showed that factors involved in the interaction vary mostly among different species and strains, mainly regarding interaction between bacterial adhesins and extracellular matrix or mucus proteins. We have investigated the adhesive properties of *Lactobacillus plantarum*, a member of the human microbiota of healthy individuals.

**Results:**

We show the identification of a *Lactobacillus plantarum *LM3 cell surface protein (48 kDa), which specifically binds to human fibronectin (Fn), an extracellular matrix protein. By means of mass spectrometric analysis this protein was identified as the product of the *L. plantarum enoA1 *gene, coding the EnoA1 alfa-enolase. Surface localization of EnoA1 was proved by immune electron microscopy. In the mutant strain LM3-CC1, carrying the *enoA1 *null mutation, the 48 kDa adhesin was not anymore detectable neither by anti-enolase Western blot nor by Fn-overlay immunoblotting assay. Moreover, by an adhesion assay we show that LM3-CC1 cells bind to fibronectin-coated surfaces less efficiently than wild type cells, thus demonstrating the significance of the surface displaced EnoA1 protein for the *L. plantarum *LM3 adhesion to fibronectin.

**Conclusion:**

Adhesion to host tissues represents a crucial early step in the colonization process of either pathogens or commensal bacteria. We demonstrated the involvement of the *L. plantarum *Eno A1 alfa-enolase in Fn-binding, by studying LM3 and LM3-CC1 surface proteins. Isolation of LM3-CC1 strain was possible for the presence of expressed *enoA2 *gene in the *L. plantarum *genome, giving the possibility, for the first time to our knowledge, to quantitatively compare adhesion of wild type and mutant strain, and to assess doubtless the role of *L. plantarum *Eno A1 as a fibronectin binding protein.

## Background

The role of a balanced human gut microbiota is crucial in host health, representing a protection against disease and a support for efficient and healthy gut function [1-3]. The microbial species composition varies along the length of the gut, and it is influenced by diet, environment, and aging [4]. The protective role of commensal bacteria within the gut consists in outcompeting invading pathogens for ecological niches and metabolic substrates [5,6]. In particular, some indigenous bacteria are believed to have the ability to overcome pathogens by producing acids, bacteriocins or hydrogen peroxide. Moreover, it is now believed that interference with pathogen adhesion could be a powerful way of preventing infection [7]. The gut microbiota represents also an important modulator of the immune system, educating the infant immune system, and being a source of non-inflammatory immune stimulators in healthy individuals [8,9].

Lactic acid bacteria (LAB) of the genus *Lactobacillus *and *Bifidobacterium *are one of the most important health promoting groups of the human intestinal microbiota. Traditionally present in the dairy products, such microorganisms have been used for treatment and prevention of gut diseases since long time ago, and more recently the beneficial effects of some probiotic LAB strains were assessed by clinical trials [7]. Specific probiotic LAB strains were also shown to modulate the host immune system and to reduce allergic symptoms. For these reasons they are considered good live vectors for vaccine delivery [10,11].

*Lactobacillus plantarum *is a member of the human microbiota of healthy individuals [12]. Due to its metabolic versatility, and to its strong ability to preserve food and prevent spoilage, *L. plantarum *has been largely used as starter in food industry and for the development of probiotic food [13,14]. The probiotic features of many strains of *L. plantarum *have been extensively studied and well assessed. Among the features necessary to provide health benefits, probiotic microorganisms must have the ability to adhere to human intestinal cells and consequently to colonize the gut. Some strains of *L. plantarum *have been positively tested for their ability to adhere to human colonic cell lines, to survive gastrointestinal passage and to persist in the intestine of healthy volunteers after oral administration [15-17].

Pathogen and commensal bacteria have evolved many mechanisms functional to a successful colonization of the host gut: rapid multiplication, expression of adhesins, and the use of non-specific adhesion mechanism like hydrophobicity, electrostatic interactions and protective capsules [18]. Studies on mechanisms mediating adhesion of lactobacilli to human intestinal cells showed that factors involved in the interaction vary mostly among different species and strains, mainly regarding interaction between bacterial adhesins and extracellular matrix (ECM) or mucus proteins [19,20]. Adhesion of *L. plantarum *to human intestinal cell line HT-29 is mediated by a mannose-specific adherence mechanism [21,22]. This kind of adherence mechanism is common among Gram-negative bacteria, but rare for Gram-positive. This could be the reason for the ability of *L. plantarum *to compete with potentially pathogenic microorganisms for receptors on the surface of human intestinal ECM. The mannose specific adhesin Msa of *L. plantarum *has been identified by *in silico *matching of genotypic and phenotypic characteristics of *L. plantarum *strains showing positive or negative mannose adhesion capability [23]. In the *L. plantarum *WCFS1 genome sequence, the corresponding *msa *gene was annotated as a gene coding a putative cell surface protein, supporting the finding that the Msa protein is an adhesin [24].

Fibronectin (Fn) is an ECM protein shown to be involved in the bacteria-endothelial cell interaction and several Fn-binding proteins have been identified in Gram positive bacteria. The aim of this study was the characterization of Fn-binding proteins in the adhesive probiotic species *L. plantarum*. In our study we selected the *L. plantarum *LM3 strain, previously classified as a mannose adhesive strain [23].

## Results

### Identification of L. plantarum Fn-binding surface proteins

**Figure 1:**
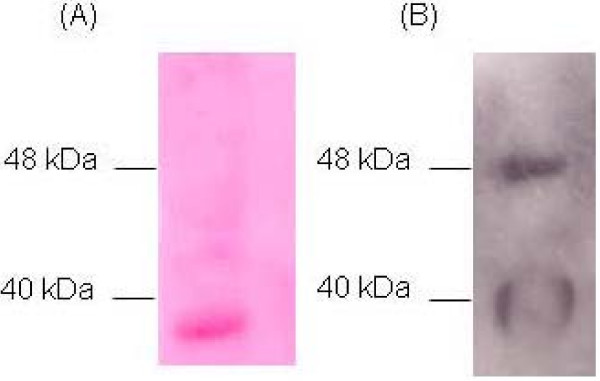
**Identification of Fn-binding surface proteins of *L. plantarum *LM3**. (A) PVDF membrane stained with Ponceau S. (B) Autoradiography of PVDF membrane after peroxidase assay.

We classified the *L. plantarum *LM3 as an adhesive strain, according to Jacobsen and coworkers [25], by binding assay performed on Caco-2 cells. To identify putative bacterial adhesins, we performed immunoblot overlay assays of LM3 surface proteins with human fibronectin. The LM3 surface proteins were extracted, separated by SDS-PAGE, blotted onto a PVDF membrane, and tested for fibronectin binding. Two signals, corresponding to proteins with an apparent molecular mass of 40 kDa and 48 kDa, were detected (Fig. 1). The putative fibronectin binding proteins were subjected to in-gel tryptic digestion and analyzed by MALDI-TOF mass spectrometry. Protein identification was achieved by database searches using the Mascot search program. Results are listed in Table 1. The MALDI-TOF-MS analysis performed on the 40 kDa putative adhesin of the LM3 strain revealed that the band contained five co-migrating proteins: D-alanyl transfer protein DltD; GTP cyclohydrolase II; oligopeptide ABC transporter, ATP-binding protein; alfa-subunit of the E1 component of the pyruvate dehydrogenase complex; ATP-binding protein of the glycine betaine/carnitine/choline ABC transporter (Table 1). The 48 kDa protein was identified as the *L. plantarum *WCFS1 *enoA1 *gene product, namely the phosphopyruvate hydratase or alfa-enolase (EnoA1). A database search in the *L. plantarum *WCFS1 genome, a strain showing a high identity percentage with the LM3 genome [26], showed that *enoA1 *is the last gene of the pentacistronic *cggR *operon containing genes encoding the central glycolytic enzymes [24].

**Table 1:** Identification of fibronectin binding proteins by MALDI-TOF-MS analysis.

Sample	Acc. N.	Protein	MW (kDa)	Gene
Band A (48 kDa)	GI_28377645	Phosphopyruvate hydratase (enolase)	48,057	*enoA1*

Band B (40 kDa)	**GI_28378651**	D-alanyl transfer protein DltD	48,633	*dltD*

	GI_28378156	GTP cyclohydrolase II	43,873	*ribA*

	GI_28378767	Pyruvate dehydrogenase complex, E1 component, alfa subunit	41,443	*pdhA*

	GI_28378030	Oligopeptide ABC transporter, ATP-binding protein	39,760	*oppD*

	GI_28378307	Glycine betaine/carnitine/choline ABC transporter, ATP-binding protein	44,505	*opuA*

### Sub-cellular localization of the L. plantarum alfa-enolase

**Figure 2:**
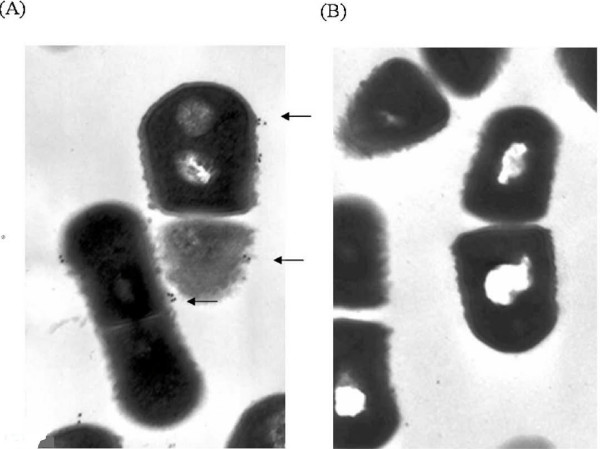
**Immunogold staining of enolase on *L. plantarum *LM3 thin sections**. The enolase was detected on the bacterial surface by anti-Eno antibodies and 10 nm-colloidal gold-labeled secondary antibody. LM3 with (A) and without (B) primary antibody. The 10-nm gold particles are indicated by the arrows.

To prove the surface localization of the EnoA1 protein, immune electron microscopy experiments were performed on whole *L. plantarum *LM3 cells, using an anti-enolase polyclonal antibody generated against the *Streptococcus pneumoniae *alfa-enolase, sharing 71% identity with the EnoA1 protein. Bacterial cells were incubated with anti-enolase antibody, then treated with secondary antibody conjugated with 10 nm-colloidal gold particles and embedded in Araldite M resin. The pellets were sliced using an ultramicrotome and observed by a scanning electron microscope. Electron microscopy observation revealed the presence of gold particles on the surface of *L. plantarum *LM3 cells, thus indicating the presence of a alfa-enolase on the cell wall of whole cells (Fig. 2).

### Isolation of the LM3-CC1 strain

The *L. plantarum *WCFS1 genome, sharing high percent of sequence identity with the LM3 strain [26], is characterized by the presence of two genes (lp_0792 and lp_1920) encoding for two putative proteins with predicted alfa-enolase activity [24]. Both genes, named *enoA1 *and *enoA2*, are expressed in *L. plantarum *LM3 under standard growth conditions as detected by microarray analysis (Muscariello, personal comunication). The *enoA1 *gene belongs to the so-called "central glycolytic genes operon" (*cggR*), and is located at the 3' end of the operon [24]. The presence of two expressed *eno *genes allowed the isolation of a *L. plantarum *strain carrying a null mutation in the *enoA1 *gene, by a double-step homologous recombination process. The entire *enoA1 *coding region, with the exception of the first 109 bp, was replaced by a DNA fragment carrying the *ery *antibiotic resistance cassette. Sixteen percent of the recombinant clones analyzed showed that a double homologous recombination event had occurred, as detected by PCR analysis. One of these clones, hereby named LM3-CC1, showing a doubling time comparable to its isogenic wild type strain, was chosen for further analysis.

### Transcriptional analysis of the cggR operon in the LM3-CC1 strain

**Figure 3:**
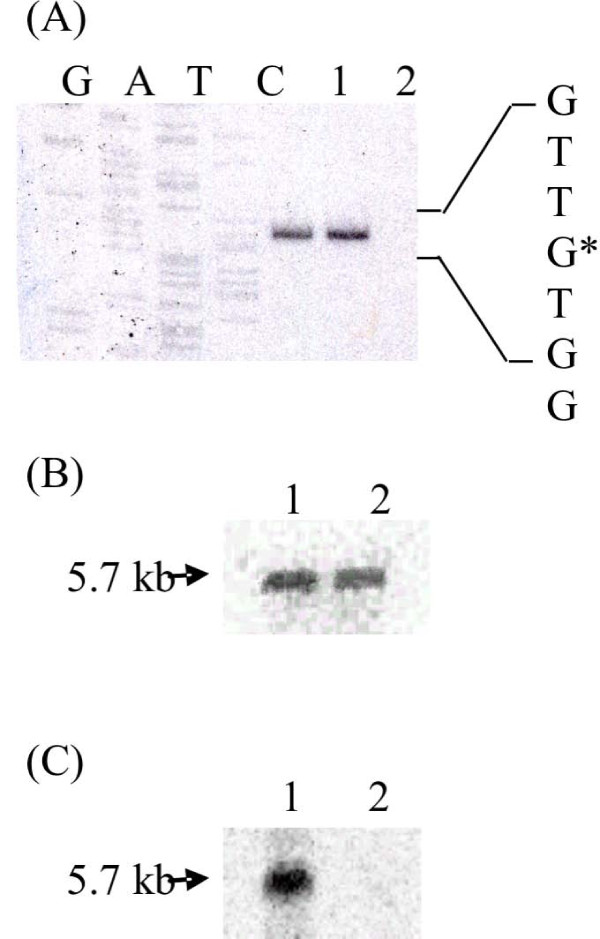
**Transcriptional analysis of the *L. plantarum cggR *operon**. (A) Primer extension products were obtained by using oligonucleotide cgg4 and total RNA extracted from LM3 and LM3-CC1. As a reference, sequencing reactions were performed with the same primer. Lanes: 1, LM3; 2, LM3-CC1. Northern blot hybridization of total RNA extracted from LM3 and LM3-CC1 with *cggR *(B) or *enoA1 *(C) probes. Lanes: 1, LM3; 2, LM3-CC1.

In order to verify transcription of the *cggR *operon in the mutant strain, a primer extension analysis was performed on total transcripts extracted from LM3 and LM3-CC1 strains. No difference was detected in the two strains, as measured by PhosphorImager (Fig. 3, panel A), showing that replacement of the *enoA1 *gene with the *ery *antibiotic resistance cassette did not affect transcription of the operon. To further analyze the effect of the *enoA1 *gene deletion on transcription of the *cggR *operon, Northern blot analysis was performed on total RNA extracted from both strains. A 360-bp fragment internal to the *cggR *gene, the first gene of the pentacistronic operon, was used as a probe. A signal of about 5.7 kb, corresponding to transcripts from the entire *cggR *operon, was observed in both LM3 and LM3-CC1 strains (Fig. 3, panel B). The difference of calculated size between *cggR *transcripts from the wild-type and the mutant strain, less than 200 bp, is likely to be undetectable on a 1% agarose gel. When a different probe was used (320-bp fragment internal to the *enoA1 *gene), the 5.7 kb signal was observed in the LM3 strain, whereas no signal was detected in the LM3-CC1 strain (Fig. 3, panel C).

### Analysis of the LM3-CC1 cell surface proteins

**Figure 4:**
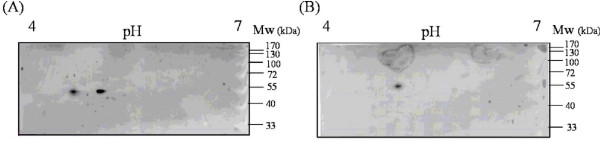
**Western blot analysis of *Lactobacillus plantarum *LM3 (A) and LM3-CC1 (B) surface proteins resolved in a two-dimensional gel electrophoresis, and assayed with anti-streptococcal enolase antibody**.

Surface proteins extracted from wild type and LM3-CC1 cells were resolved by two dimensional electrophoresis, and western blotted by using the anti-enolase polyclonal antibody generated against the *Streptococcus pneumoniae *alfa-enolase. In the wild type strain the presence of two proteins, showing molecular mass of 48 and 46,6 kDa, and p*I *of 4,6 and 4,9 respectively, was detected (Fig. 4, panel A), corresponding to EnoA1 and EnoA2 as reported in Swiss-Prot data base. The same analysis, performed on LM3-CC1 surface proteins, showed the presence of EnoA2 (Fig 4, panel B), while EnoA1 was not anymore detected.

**Figure 5:**
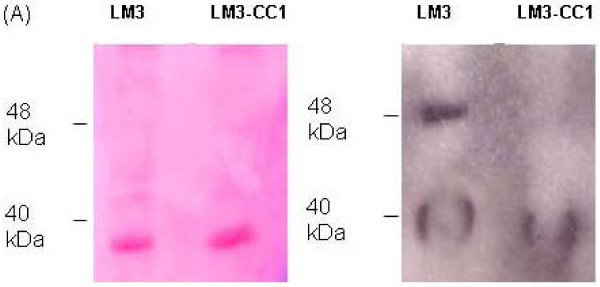
**Fibronectin overlay assay performed on surface proteins of *L. plantarum *LM3 and LM3-CC1**. (A) PVDF membrane stained with Ponceau S. (B). Autoradiography of PVDF membrane after peroxidase assay. Lanes: 1, LM3; 2, LM3-CC1.

Fn-overlay assay was performed on cell surface proteins extracted from wild type and LM3-CC1 cells (Fig. 5). A positive signal corresponding to a 40 kDa Fn-binding protein was detected in cell surface protein extracts of both strains (Fig. 5, panel B), while the signal corresponding to the 48 kDa adhesin, namely the EnoA1 protein, was detected only in the wild type strain.

### Adhesion of wild type and mutant strains to immobilized fibronectin

**Figure 6:**
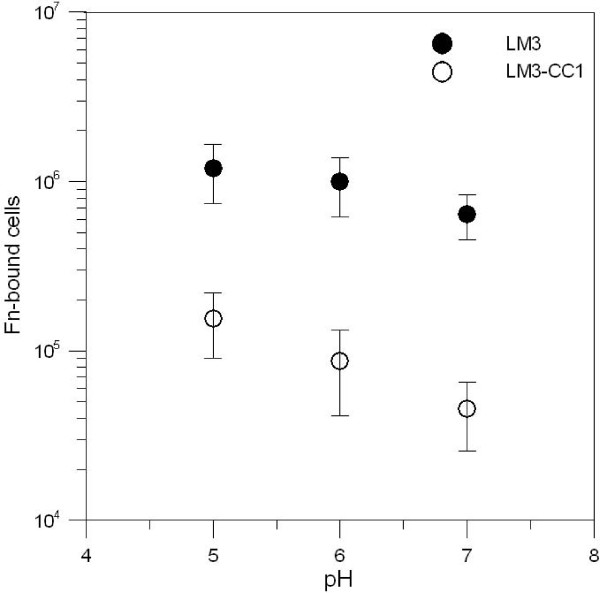
**Binding of LM3 and LM3-CC1 to fibronectin immobilized on microtiter plate wells. Adhesion of both strains was evaluated by Real-time PCR**. Adhesion is given as number of bacteria bound to fibronectin at different values of pH. Error bars represent ± standard deviation of the mean values.

In order to prove the significance of the 48 kDa surface protein for *L. plantarum *LM3 adhesion to fibronectin, the extent of binding of LM3 and LM3-CC1 cells was analyzed on Fn-coated microtiter plates. To verify if pH could affect the binding, experiments were repeated with bacteria cells pre-treated in Tris buffers at pH ranging from 5.0 to 7.0. Bacterial adhesion was evaluated by quantification of Fn-bound cells by Real-time PCR, amplifying 16S ribosomal DNA with species-specific primers [27]. A significant difference in adherence (*P *< 0.05) was observed, with about ten times more wild type than mutant cells adhering to fibronectin (Fig. 6). Nevertheless, no significant difference in adherence was found upon pre-treatment of both strains in different pH buffers.

## Discussion

Microorganisms have developed different mechanisms to successfully colonize human tissues, in terms of either pathogenic or commensal type of interaction [1,18]. The adhesion to host tissues represents a crucial early step in the colonization process, and bacterial pathogens usually express surface-bound adhesion molecules interacting with host receptors. For instance, in early stages of colonization, bacteria may use pili, fimbria and a variety of adhesins to interact with a plethora of different host elements displayed on the host cell surface, either membrane-bound or secreted, including ECM proteins. Among the ECM, the glycoprotein fibronectin was shown to be the target for interaction with many pathogens as well as with some probiotic bacteria [19,28-34]. *L. plantarum *is a lactic acid bacterium often described as an inhabitant of the human intestinal tract. The probiotic features of some strains of *L. plantarum *have been extensively described. A mannose specific adhesion mechanism for interaction of *L. plantarum *299 with human colonic cell line HT-29 was described many years ago [21], and more recently the identification of the mannose-specific adhesin was shown [23]. In this report we investigated *L. plantarum *fibronectin binding proteins. To this aim, we used the *L. plantarum *LM3 strain, which we classified as an adhesive strain on Caco-2 cell line according to Jacobsen and coworkers [25]. This strain was also previously classified as a mannose adhesive strain [23]. In particular, we describe the identification of a *L. plantarum *cell surface displayed protein, of apparent molecular mass of 48 kDa, which specifically binds to human fibronectin. By means of mass spectrometric analysis the 48 kDa Fn-specific adhesin was identified as the EnoA1 alfa-enolase, encoded by the *enoA1 *gene. The cell surface localization of the protein was proved by immune electron microscopy using a polyclonal antibody generated against the *Streptococcus pneumoniae *alfa-enolase. Moonlighting proteins are defined as highly conserved cytoplasmatic proteins that, when expressed on the bacterial cell wall, acquire a "moonlighting" function different from their well-known activity performed in the cytoplasm. Moonlighting functions for many housekeeping enzymes, such as enolase, is now well documented, and some of these enzymes may act as virulence factor for pathogens [35,36]. Indeed, surface-displayed enolase of different pathogen bacteria binds to host glycoprotein: in *Staphylococcus aureus *alfa-enolase was shown to bind the ECM protein laminin [37]; surface-displayed enolase was demonstrated to bind to plasminogen in *Streptococcus pneumoniae *and *Bacillus anthracis *and to mucin and plasminogen in *Streptococcus mutans *[38-40]. Moreover, surface-displayed enolase was demonstrated to bind to plasminogen also in bacterial species of the human indigenous microbiota [41,42].

To assess the role of EnoA1 as a *L. plantarum *Fn-specific adhesin, we isolated the *L. plantarum *LM3-CC1 strain, carrying a null mutation in the *enoA1 *gene. Disruption of the *eno *gene was unsuccessful in *S. pneumoniae*, demonstrating that alfa-enolase is essential for viability of this microorganism [38]. The isolation of the LM3-CC1 mutant strain was planned upon the observation that in the *L. plantarum *WCFS1 genome, sharing high percent of sequence identity with the LM3 strain [26], the *enoA2 *gene, coding for EnoA2 alfa-enolase, is also present and is expressed under standard growth conditions (Muscariello, personal comunication). By Western blot analysis of cell surface proteins resolved on two dimensional gel, both alfa-enolases were detected in the wild type, while only EnoA2 was shown to be present in the LM3-CC1 surface protein extracts. Moreover, immunoblotting assays performed on the LM3-CC1 strain clearly demonstrated the lack of the Fn-specific EnoA1 adhesin. The characterization of the LM3-CC1 mutant strain shows that EnoA1 contributes significantly to adhesion to fibronectin. In fact the mutant strain adheres less efficiently than wild type strain to Fn-coated wells. Residual adhesion of LM3-CC1 is probably due to other adhesion molecules involved in the interaction bacteria-ECM proteins. The possibility of a comparative analysis between the LM3 wild type and LM3-CC1 mutant strain doubtless allows to assess the role of *L. plantarum *Eno A1 as a fibronectin binding protein.

## Conclusion

We demonstrated the surface localization of the *L. plantarum *EnoA1 alfa-enolase, which specifically binds to human fibronectin, an extracellular matrix protein. In *L. plantarum*, the expression of two *eno *genes, as detected by microarray analysis, allowed the isolation of the LM3-CC1 strain, carrying a null mutation in the *eno*A1 gene. Comparative analysis of the LM3 and LM3-CC1 surface proteins by anti-enolase Western blot, and by Fn-overlay immunoblotting assay, doubtless demonstrated the involvement of the EnoA1 protein in binding of *L. plantarum *to fibronectin. Moreover, by an adhesion assay we showed that LM3-CC1 cells bind to fibronectin-coated surfaces less efficiently than wild type cells. The isolation of the LM3-CC1 strain gave the possibility, for the first time to our knowledge, to quantitatively compare adhesion of wild type and mutant strain, thus demonstrating the significance of the surface displaced EnoA1 protein in the mechanism of adhesion to fibronectin.

## Materials and methods

### Bacterial strains and culture conditions

*L. plantarum *LM3 [43], *L. plantarum *LM3-CC1 (this study), and the *Escherichia coli *Top10 strains were used throughout this study. The *L. plantarum *strains were cultured at 30°C in MRS broth supplemented with either 0.5% or 2% glucose. The *E. coli *strain was grown at 37°C in TY broth. When needed, the following antibiotics were added: erythromycin 250 microgramms/ml for *E. coli*, and 5 microgramms/ml for *L. plantarum*; lincomycin 10 microgramms/ml for *L. plantarum*.

### SDS-PAGE and fibronectin overlay assay

Surface proteins (10 microgramms), obtained by ultracentrifugation of French-pressed *L. plantarum *LM3 cells, were subjected to SDS-PAGE (10%). The proteins were transferred to an Immun-Blot PVDF membrane (BIO-RAD Inc.) using the Mini Trans-Blot equipment (BIO-RAD Inc.), at 35 V, 4°C, for 16 h in Towbin Buffer (20 mM Tris base, 192 mM glycin, 20% methanol, pH 8.3). The PVDF membrane was reversibly stained with Ponceau S (Sigma). To detect fibronectin binding, overlay assays were performed as described [28] with some modifications: the PVDF membranes were incubated with blocking buffers I and II, washed with NN' buffer and overlaid with 0.2 microgramms/ml of human fibronectin (Calbiochem-Oncogene) overnight at 4°C. After three washes, primary antibody for fibronectin (anti-human IgG, mouse generated, Calbiochem-Oncogene), diluted 1:60000 in NN' buffer, was added and incubated at r.t. for 1 h. The PVDF membranes were washed three times and incubated with a 1:5000 dilution of the secondary antibody conjugated with horseradish peroxidase (GE Healthcare), in blocking buffer, at r.t. for 1 h. After three washes the bound antibodies were revealed with ECL PLUS kit (GE Healthcare).

### Protein identification by Peptide Mass Fingerprinting

In-gel digestion was carried out according to Shevchenko and co-workers [44]. Briefly, selected Comassie-stained protein bands were excised from gels and destained with 50% acetonitrile (ACN) in 100 mM ammonium bicarbonate, dehydrated in ACN and vacuum-dried in a Speed-Vac centrifuge. Proteins contained in gel pieces were treated with 10 mM dithiothreitol in 100 mM ammonium bicarbonate at 56°C for 1 h to reduce disulphide bridges, and alkylation of the cysteine residues was carried out with 50 mM iodoacetamide in 100 mM ammonium bicarbonate at r.t. in the dark for 30 min. Gel bands were dehydrated in ACN and re-swollen in 10 ml of buffer solution (25 mM ammonium bicarbonate pH 8.4) containing 10 ng/ml of trypsin at 4°C for 15 min. Excess of enzymatic solution was removed and 20 ml of buffer solution were added to the gel pieces. Digestion proceeded overnight at 37°C. The obtained peptide mixture (0.5 ml) was mixed with 0.5 ml of a saturated solution of αlfa-cyano-4-hydroxycinnaminic acid [10 mg/ml in 50% ACN containing 25 fmol/microliter angiotensin and 125 fmol/microliter ACTH (Adrenocorticotropic Hormone fragment 18–39) as internal standards], spotted directly on a MALDI target plate and dried under ambient condition. All mass spectra were generated on a MALDI-TOF mass spectrometer Voyager DE™ PRO (Applied Biosystems), operating in positive-ion reflectron mode. The laser intensity (N_2_, 337 nm) was set just above the ion generation threshold and pulsed every 10 ns. Mass spectra were acquired from each sample in the 700–3500 m/z range, by accumulating 100 laser shots and were calibrated using as internal standards the monoisotopic peaks of angiotensin (m/z 931.5154) and ACTH (m/z 2465.1989). All mass values are reported as monoisotopic masses. Protein identification was achieved by using the MALDI mass spectral data for database searches against the NCBInr database using the MASCOT search algorithm . Parameters for all searches were as follows: bacteria as taxonomic category, trypsin as enzyme, carbamidomethyl as fixed modification for cysteine residues and methionine oxidation as variable modification, one missing cleavage and 30 ppm as mass tolerance for the monoisotopic peptide masses.

### Resolution of Lactobacillus cell wall proteins by 2DE

Five hundred micrograms (wet wt) of cell wall material, obtained by ultracentrifugation of French-pressed *L. plantarum *LM3 cells, was suspended in 100 micro-liters of 100 mM Tris-HCl, pH 7.4, containing 1% SDS and boiled for 10 min. After cooling, 30 micro-liters solubilization buffer, consisting of 7 M urea, 2 M thiourea, 4% CHAPS, 1% DTT, 0.2% Bio-Lytes pH 3–10, and 0.002% bromophenol blue, was added. The mixture was gently agitated for 30 min at room temperature, followed by TCA precipitation. The cell wall fraction was solubilized in IEF solution containing 7 M urea, 2 M thiourea, 4% CHAPS, 1% DTT, 1% Bio-lytes 3–10 and 0.002% bromophenol blue for two-dimensional polyacrylamide gel electrophoresis (2DE) analysis. Two-dimensional electrophoresis of extracted cell wall proteins was performed as follows; in the first dimension, isoelectrofocusing (IEF) was performed using the Bio-Rad PROTEAN IEF cell and Ready linear IPG strips (7 cm, pH 3–10). The IPG strips were hydrated overnight (16 h) with 2 micrograms of membrane protein and IEF was carried out at 500 V, rapid mode, for 30 min, 1000 V for 30 min, followed by gradient voltage to 8000 V in 30 min, 8000 V for 1 h, 50 V until stop. Prior to electrophoresis in the second dimension, IPG strips were thawed and incubated for 10 min in 2 ml equilibration buffer containing 6 M urea, 1,5 M Tris-HCl, pH 8.8, 2% SDS, 20% glycerol, and 2% DTT, followed by incubation with equilibration buffer supplemented with 2.5% iodoacetamide for 10 min at room temperature. Second-dimensional 12% polyacrylamide gels were run at 160 V for 2.5 h.

### Western blot analysis

*L. plantarum *LM3 cell wall proteins (2 micrograms) were analyzed by 2DE as reported above and blotted onto a nitrocellulose membrane (Hybond ECL, Amersham), using the Mini Trans-Blot equipment (BioRad Inc.), at 90 mA, 4°C, for 16 h in Towbin Buffer. The membrane was incubated in TBST buffer (20 mM Tris base, 137 mM NaCl, 0.15% Tween 20) + nonfat dry milk 4% for 1 h at r.t., with orbital shaking, then incubated with anti-streptococcal enolase (IgG, rabbit generated), diluted 1:1000 in blocking buffer, at r.t. for 1 h. After three washes with TBST the membrane was incubated with a 1:10000 dilution of the secondary antibody conjugated with horseradish peroxidase (GE Healthcare), in blocking buffer at r.t. for 1 h. After three washes with TBST the bound antibodies were revealed with ECL PLUS kit (GE Healthcare).

### Immune electron microscopy

*L. plantarum *LM3 cells were grown in 10 ml of MRS broth with 2% glucose up to stationary phase, centrifuged, washed three times with phosphate-buffered saline (PBS) buffer, and resuspended in the same buffer to a concentration of 10^9 ^cells/ml. One ml of the cellular suspension was centrifuged and resuspended in 95 micro-liters of PBS with 1% bovine serum albumin (BSA). The anti-streptococcal enolase primary antibody, diluted 1:250 or 1:500, was added and incubated for 30 min at r.t with shaking. The cells were washed twice with PBS, added with 1% BSA, and incubated with 25 micro-liters of a 1:5 dilution of the secondary antibody conjugated with 10 nm-colloidal gold particles (AuroProbe, Amersham), in PBS-BSA, for 30 min at r.t. with shaking. The cells were then washed with PBS-BSA; the last washing was performed with PBS only. The pellet was fixed for 4 h at 4°C with 2.5% glutaraldehyde in 0.1 M cacodylate buffer (CB) and washed overnight with 0.15 M CB. The pellet was treated with 1% OsO_4_in 0.1 M CB for 1 h at 4°C, then washed twice in 0.15 M CB. The pellet was treated with increasing alcohol concentrations (30%, 50%, 70%, 96%, 100%), with propylene oxide, 2/3 propylene oxide + 1/3 Araldite M resin, 1/3 propylene oxide + 2/3 resin, and embedded overnight in pure resin at 60°C. The embedded pellet was cut with an ultramicrotome and the slices were observed with an electron microscope.

### Construction of a strain carrying a null mutation in the enoA1 gene

Based on the high percent of identity between the LM3 strain and the WCFS1 strain [26]. DNA fragments were amplified from the LM3 chromosome using the WCFS1 sequence data. The chromosomal DNA of *L. plantarum *LM3 was used as a template for two PCR reactions in order to amplify the two fragments UP (830 bp) and DOWN (930 bp). UP includes the *tpiA *gene and the first 109 bp of the *enoA1 *gene. DOWN corresponds to the lp_0793 locus. In the PCR reaction the oligonucleotides eno1 (5'-CGCGAATTCCCCAGCCCTTTTCTTACAGG-3') and eno2 (5'-CGCGGATCCCGATACCGCGGCCAAATGCG-3') were used to amplify the UP region, and eno5 (5'-AAAACTGCAGGGAGCGAGCAGAATGGATGAC-3') and eno6 (5'-CCCAAGCTTGCAATGTGCGGCCGCTGTGC-3') were used to amplify the DOWN region. The UP and DOWN fragments were cloned, respectively, upstream and downstream of the *ery *cassette of pUC18Ery plasmid, in the *Eco*RI-*Bam*HI and *Pst*I-*Hin*dIII sites, yielding the pCV3 plasmid, used to transform *L. plantarum *as described [43].

### Primer extension and Northern blot analysis

Total RNA from *L. plantarum *cells grown to mid-exponential phase on MRS medium supplemented with 2% glucose was isolated as described [43]. Primer extension products of *cggR *transcript were obtained by using oligonucleotide cgg4 (5'-CGACGACTCAGCATGTCAAC-3'). Northern blotting on total RNA extracted from cells grown on glucose, using 360- and 320-bp internal fragments of the *cggR *and *enoA1 *genes as probes, was performed as described [43].

### Adherence of bacteria to Fn-coated surfaces

For adherence assays, *Lactobacillus plantarum *LM3 was grown in MRS broth with 0,5% glucose for 12 h at 30°C. Cells were harvest by centrifugation at 3500 rpm for 15 minutes at 4°C for three times and the pellets were resuspended in 50 mM Tris-HCl at pH 5, 6, 7, 8 and incubated at 30°C for 1 h. Cells were harvest by centrifugation and resuspended in Dulbecco's modified Eagle's medium (DMEM) with 2% fetal bovine serum (FBS, Invitrogen).

Microtiter plates (96 wells) were coated overnight with fibronectin 0,25 micrograms/well at 4°C overnight and subsequently blocked with 2% BSA for 1 h at 37°C. After three washes with PBST a 100 micro-liters bacterial suspension containing 5 × 10^7 ^CFU in DMEM was added, and after 2 h of incubation at 37°C the wells were washed three times with PBST. Adherent bacteria were detached from the wells by adding 100 micro-liters of 10% trypsin and quantified by real-time PCR. An aliquot of 20 micro-liters was transferred into a 0.2 ml-reaction tube and incubated for 10 min at room temperature with 3.8 micro-liters of trypsin inhibitor solution (Type I-S: from soybean, Sigma; 1 mg/ml in H_2_O). The bacterial cells were specifically quantified by real-time PCR performed with species-specific primers: Bact-0011f (5'-AGAGTTTGATCATGGCTCAG-3') and Lab-0677r (5'-CACCGCTACACATGGAG-3') [27]. Real-time PCR was performed in a LightCycler instrument (Roche, Mannheim, Germany) and SYBER Green I fluorophore was used to correlate the amount of PCR product with the fluorescent signal. Amplification was carried out in a 20 micro-liters final volume containing 2 micro-liters of cell suspension, 0.5 micromolar of each primer and 4 micro-liters of LightCycler-FastStart DNA Master SYBR Green I (Roche). The experimental protocol consisted of the following programs: (i) starting preincubation at 95°C for 10 min; (ii) amplification including 30 cycles of 4 steps each at the temperature transition time of 20°C/s: denaturation at 95°C for 15 s, annealing at 63°C for 25 s, extension at 72°C for 30 s, and fluorescence acquisition at 85°C for 5 s; (iii) melting curve analysis: heating at 20°C/s to 95°C; cooling at 20°C/s to 60°C with 15 s hold, and then heating 0.1°C/s until 99°C. As internal standards we amplified serial dilutions of the respective bacteria in PBS ranging from 2 × 10^6 ^to 2 × 10^3 ^CFU/αl.

## Competing interests

The authors declare that they have no competing interests.

## Authors' contributions

CC and VV performed most of the experiments; RAS performed the MALDI-TOF analysis; MC performed the Western blot analysis with anti-streptococcal enolase; MV performed the immune electron microscopy; LM performed transcription analysis in the LM3-CC1 strain; RM contributed to experiment design and discussion; MS contributed in discussions during the work and in the preparation of the manuscript. All authors read and approved the final manuscript.
